# Event-Based Computation for Touch Localization Based on Precise Spike Timing

**DOI:** 10.3389/fnins.2020.00420

**Published:** 2020-05-19

**Authors:** Germain Haessig, Moritz B. Milde, Pau Vilimelis Aceituno, Omar Oubari, James C. Knight, André van Schaik, Ryad B. Benosman, Giacomo Indiveri

**Affiliations:** ^1^Institute of Neuroinformatics, University of Zurich and ETH Zurich, Zurich, Switzerland; ^2^International Centre for Neuromorphic Systems, MARCS Institute, Western Sydney University, Penrith, NSW, Australia; ^3^Max Planck Institute for Mathematics in the Sciences, Leipzig, Germany; ^4^Max Planck School of Cognition, Leipzig, Germany; ^5^Institut de la Vision, Sorbonne Université, Paris, France; ^6^Centre for Computational Neuroscience and Robotics, School of Engineering and Informatics, University of Sussex, Brighton, United Kingdom; ^7^University of Pittsburgh, Pittsburgh, PA, United States; ^8^Carnegie Mellon University, Pittsburgh, PA, United States

**Keywords:** temporal coding, event-based sensors, spatio-temporal patterns, spike-based computing, touch localization

## Abstract

Precise spike timing and temporal coding are used extensively within the nervous system of insects and in the sensory periphery of higher order animals. However, conventional Artificial Neural Networks (ANNs) and machine learning algorithms cannot take advantage of this coding strategy, due to their rate-based representation of signals. Even in the case of artificial Spiking Neural Networks (SNNs), identifying applications where temporal coding outperforms the rate coding strategies of ANNs is still an open challenge. Neuromorphic sensory-processing systems provide an ideal context for exploring the potential advantages of temporal coding, as they are able to efficiently extract the information required to cluster or classify spatio-temporal activity patterns from relative spike timing. Here we propose a neuromorphic model inspired by the sand scorpion to explore the benefits of temporal coding, and validate it in an event-based sensory-processing task. The task consists in localizing a target using only the relative spike timing of eight spatially-separated vibration sensors. We propose two different approaches in which the SNNs learns to cluster spatio-temporal patterns in an unsupervised manner and we demonstrate how the task can be solved both analytically and through numerical simulation of multiple SNN models. We argue that the models presented are optimal for spatio-temporal pattern classification using precise spike timing in a task that could be used as a standard benchmark for evaluating event-based sensory processing models based on temporal coding.

## 1. Introduction

Information transmission in neural networks is often described in terms of the rate at which neurons emit action potentials. Neurons are typically assumed to encode values—such as the orientation of a bar—using their mean firing rate, with individual spikes emitted using a Poisson process (Dean, 1981). Neurons in higher processing areas of the brain (e.g., in primary visual cortex) have been shown to demonstrate variable spike timing in response to repetitions of identical stimuli (Hubel and Wiesel, 1962). This variability is commonly interpreted as being the result of noise (or noisy background activity) which can be assumed to be an additive signal to the sensory input one (Baudot et al., 2013). This linear separation of signal and noise has been used to justify rate- and/or population-coding by averaging across time and/or neuronal populations (Shadlen and Newsome, 1998; Dayan and Abbott, 2001). These observations led to the common assumption that the main mode of information transmission in most brain areas is encoded in the neurons average spike-frequency. This assumption, supported by many experimental investigations (Softky and Koch, 1993; Dayan and Abbott, 2001), continues to be used in the field of machine learning.

However, the time at which spikes are emitted might also carry additional information. If this is the case, the temporal-correlation of such events can then be used an extra source of information for models of computation (Dayan and Abbott, 2001; Thorpe et al., 2001). This type of signal representation is described as a *temporal code*. In the last three decades, the advantages of temporal coding have been demonstrated in computational models of fast visual processing (Thorpe et al., 2001); for the classification of time-varying signals and balance (Gütig and Sompolinsky, 2006; Deneve and Machens, 2016); in temporal interval discrimination (Buonomano and Merzenich, 1995); in state-dependent computation (Buonomano and Maass, 2009), and even for fine motor control (Laje and Buonomano, 2013). Furthermore, experimental findings have shown that the information carried in the timing of spikes can be used by the brain to discriminate textures (Hipp et al., 2006; Wolfe et al., 2008), classify temporal patterns (Mainen and Sejnowski, 1995; Wehr and Zador, 2003; Baudot et al., 2013; Goel and Buonomano, 2016) or localize an animal in its environment (O'Keefe and Recce, 1993). This evidence demonstrates that neural networks—whether biological or artificial—can use spike timing information to extract relevant cues for behavior and generate events with precise timing precision in response to time-varying input patterns (Mainen and Sejnowski, 1995).

While both rate- and temporal-codes are used to convey information in the brain, conventional ANNs, for the most part, are based only on rate-codes. The contexts and tasks in which temporal-coding can outperform rate-coding remain elusive, especially as the performance in many tasks is measured purely in terms of classification accuracy and ignores additional metrics such as latency, energy consumption and computational complexity.

In this paper, we first describe a well-constrained spatio-temporal pattern classification task inspired by the sand scorpion: localizing the source of a vibration induced by tapping on a surface, using the spatio-temporal pattern detected by an array of sensors. We then present a step-by-step analysis of conventional algorithms and five different models based on spiking neural networks for classifying the data-set of spatio-temporal patterns using both supervised and unsupervised learning rules.

## 2. Background

### 2.1. Sand Scorpion Prey Localization

**Figure 1:**
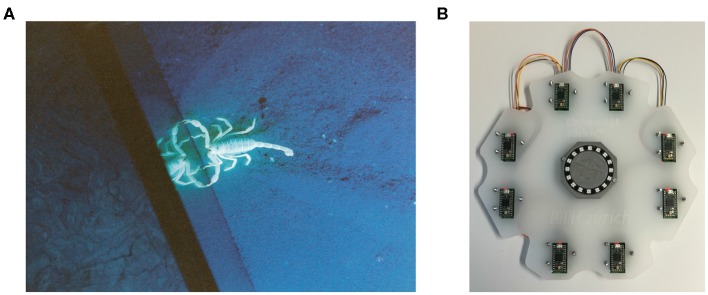
**(A)** A sand scorpion in the lab. Image courtesy of Martin Reichert and Wolfgang Stuerzl. **(B)** Our prototype.

Sand scorpions, such as the specimen shown in Figure 1A, are nocturnal predatory arachnids which, despite their primitive visual systems, can accurately locate prey such as crickets up to 50 cm away (Brownell, 1977). Brownell (1977) discovered that sand scorpions perform this feat using time-based computation based on two types of information propagated through the sand of their desert habitat: Transverse Rayleigh waves and compressional waves. Rayleigh waves travel slowly across the surface of the sand at a velocity of ≈ 50 m s^-1^ and are sensed by the scorpion's Basitarsal Compound Slit Sensilla (BCSS). Compressional waves diverge spherically from their source—traveling through the sand at ≈ 150 m s^-1^ and attenuating much more quickly than the Rayleigh waves (Brownell, 1977). Sand scorpions detect these waves using sensory hairs on their legs.

Both types of sensory organ are located on the ends of the scorpion's legs, maximizing the distance between the sensors and thus the difference in arrival time between signals measured at each one. While, theoretically, the arrival time of either type of wave could be used by the scorpion to detect the direction of its prey, Rayleigh waves travel and attenuate slower than compressional waves resulting in better range and larger time differences (1 ms rather than 0.4 ms). This intuition was supported by an ablation study in which Brownell and Farley (1979a) found that the BCSS was required for sensing direction.

As well as being able to detect the *direction* of their prey, sand scorpions can also estimate how far away it is. Brownell and Farley (1979b) suggested that the difference in amplitude of the signals received by the sensory hairs on different legs could be used to perform this computation. Here, the faster attenuation of the compressional waves is advantageous as it results in larger differences in amplitude between near and distant stimuli.

### 2.2. Computational Models of Spatio-Temporal Pattern Recognition

The ability to learn and recognize spatio-temporal sequences is a hallmark of biological neural information processing. Understanding spatio-temporal sequences is at the heart of object recognition, navigation and, in more general terms, all neuron-to-neuron communication. Each neuron receives a spatio-temporal pattern of pre-synaptic action potentials or spikes at its dendrites and sends output spikes to its post-synaptic partners. In the case of a single input channel, the problem of spatio-temporal sequence learning can be addressed by temporal coincidence detection (Carr and Konishi, 1990) or by temporal correlation detection (Krammer and Koch, 1997). The former approach provides binary outputs, whereas the latter approach provides a continuous output. In both cases, information is encoded in the timing of the incoming spike. On the other hand, if multiple input channels are present, spatio-temporal patterns can be represented by detecting coincidence or correlation of spikes arriving via the different input channels (Roy et al., 2016). Additionally, neurons have more options for capturing spatio-temporal patterns when multiple input channels are present. A neuron can use synaptic weight plasticity to emphasize certain channels over others, synaptic delay plasticity to delay certain input channels compared to others, or any combination of the two. To recognize spatio-temporal patterns, Gütig and Sompolinsky (2006) proposed the tempotron model in which synaptic weights are adjusted in a supervised manner, based on the deviation of the maximum (post-synaptic) voltage from the spiking threshold for wrongly classified patterns. Roy et al. (2016) extended the tempotron approach by using an online structural plasticity mechanism in a competitive winner-takes-all (WTA) network relying on binary synapses. Alternatively, Izhikevich (2006) proposed a learning framework in which Spike-Time Dependent Plasticity (STDP) is used to adjust synaptic weights and synaptic propagation delays are randomly sampled at the beginning of the simulation and subsequently fixed. Both approaches lead to the learning of *polychronous* neural ensembles, each encoding a different spatio-temporal pattern. Wang et al. (2013) presented a hardware implementation of polychronous networks in which propagation delays are learned in a supervised manner, based on the expected firing time of the post-synaptic neuron. Another approach to learning synaptic delays is to sample synaptic time constants from a distribution and select relevant time constants via an STDP mechanism (Gerstner et al., 1996). Thus only synapses with fitting delays which trigger post-synaptic spikes are selected. In the next sections, we will present both biological and event-based mechanisms for synaptic and neuronal plasticity to learn spatio-temporal patterns.

### 2.3. Biological Mechanisms for Synaptic Delay Plasticity

Spikes are delivered to a neuron's post-synaptic partners through its axon with a transmission delay dictated by the axon's conduction velocity. The conduction velocity is dependent on both the diameter of the axon and the thickness of the *Myelin* sheath around it (Swadlow and Waxman, 2012). Myelin is a phospholipid substance formed by glial cells and its presence increases the conduction velocity of axons by wrapping around them and acting as an electrical insulator. Furthermore, it has recently been shown that the myelination of axons can be influenced by neural activity(Markram et al., 1997; Fields, 2015; Koudelka et al., 2016) suggesting that a form of “myelin plasticity” is also at work—something that should be taken into consideration when developing learning algorithms for spiking neural networks (Baldi and Atiya, 1994; Maass, 2001).

By optimizing conduction delays, a myelin plasticity-based model opens the way to directly learning the time dynamics of incoming spikes and extracting meaningful spatio-temporal patterns. Previous conduction delay-based algorithms have not often been tested with practical tasks such as pattern recognition and clustering (Eurich et al., 1999, 2000). The DELTRON (Hussain et al., 2012) uses the tempotron model (Gütig and Sompolinsky, 2006) to adjust conduction delays through gradient descent dynamics. Paugam-Moisy et al. (2008) extended the polychronization model developed by Izhikevich (2006) to include learnable conduction delays for classification and Wang et al. (2013) applied this approach to pattern storage. Matsubara (2017) developed a probabilistic delay learning model which adjusts conduction delays and synaptic weights. However, Matsubara used this to classify time-invariant databases such as MNIST, which have no temporal structure making them a poor choice for evaluating computation based on spike timing.

### 2.4. Event-Based Spatio-Temporal Pattern Recognition

The task solved by the sand scorpion can be described more generally as spatio-temporal pattern classification and recently, two complementary approaches, specifically designed for event-based sensory signals, were proposed. Both approaches feature homogeneous and fixed synaptic time constants and adapt synaptic weights to cluster spatio-temporal patterns. In the following subsections, we detail these two approaches.

#### 2.4.1. HOTS: A Hierarchy of Event-Based Time-Surfaces

Lagorce et al. (2017) proposed an algorithm in which events are converted into a continuous-valued time surface. This approach can be understood as convolving events within a pre-defined region of interest (ROI) with an exponential decaying kernel, with the reference time being the time of the central event in the ROI. These spatio-temporal contexts are then matched to learned features using online learning, offline clustering or other methods. HOTS can be employed in a hierarchical fashion, with an increasing ROI size and time-constant for the exponential kernels and has been successfully applied to variety of classification tasks (Cohen et al., 2016; Afshar et al., 2019a).

#### 2.4.2. FEAST: Event-Based Feature Extraction Using Adaptive Selection Thresholds

To guarantee that all feature detector neurons are used equally when clustering time-surfaces, Afshar et al. (2019b) extended HOTS to feature coupled, adaptive thresholds. Every time a given feature detector emits a spike, its threshold is increased. This results in non-updated feature detectors being more likely to capture the next time-surface and means that all feature detector neurons are equally active across the data set^1^. If no feature detector captures the present time-surface, however, all thresholds are decreased. The adaptation of firing thresholds can be understood as a homeostatic plasticity mechanism (Turrigiano and Nelson, 2004; Qiao et al., 2016, 2017). In the context of continual learning, this “global”^2^ threshold adaptation might prevent convergence if unrecognized patterns are common (Afshar et al., 2014).

## 3. Methods

### 3.1. Neuromorphic Tactile Sensor Design

The problem of spatially localizing a stimulus on a 2D surface is well-defined with 5 sensors (Mahajan and Walworth, 2001; Hu and Yang, 2010). However, having an array of more than 5 sensors adds robustness to the system, so we developed the prototype shown in Figure 1 with (arbitrarily) 8 sensors. A circular configuration of the sensor array would lead to badly conditioned cases—as depicted by Mahajan and Walworth (2001)—so our 8 sensors are arranged in the non-circular manner shown in Figure 1B. An acrylic plate makes a rigid connection between the 8 sensors. As the system is statically overconstrained (5 redundant contact points), a slightly flexible acrylic plate was chosen to ensure that all 8 sensors could still touch the surface if there was any fabrication misalignment.

**Figure 2:**
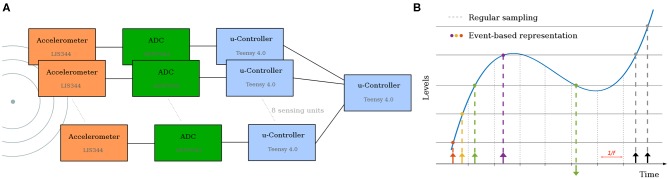
**(A)** Architecture of the electronic solution. Each accelerometer is read by its own Analog-to-Digital Converter. The local microcontroller receives the samples, applies level-crossing detection and send the master controller the spikes. **(B)** The level crossing sampling method employed, and comparison to regular sampling. In this work, only the first spike is used.

Each sensing unit consists of a Piezoelectric accelerometer for sensing vibrations and a local microcontroller-based processing unit (Teensy 4.0, ARM Cortex-M7) which reads samples from the Analog to Digital converter at 1 MHz, and then applies a level crossing detection to generate events (Astrom and Bernhardsson, 2002) (Figure 2B. All 8 sensors then transmit these events to an additional central processing unit which solves the analytical problem using the approach described in section 3.2.1 and saves the data for dataset creation (Figure 2A).

While in desert sand, a 1 ms resolution would be sufficient (Brownell, 1977), in order to work on more common mediums—which typically have faster propagation speed—we need higher temporal accuracy. Depending on the surfaces used in our experiments, a wave propagates at a speed between 200 and 300 m s^-1^, which result in a propagation time between 1 and 1.5 ms in our setup, between radially opposite sensors. However, standard accelerometers with digital output are limited to sampling rate of only a few kHz, so we decided to use an accelerometer with an analog output (*STMicro LIS344*), combined with a separate 1 MHz Analog to Digital converter (*Texas Instruments ADS7044*).

Figure 2 illustrates the architecture of the electronic solution, as well as the spike generation method. This approach of fast sampling followed by level-crossing detection was chosen for it flexibility (different encoding schemes could be tested). However, other approaches such as the one introduced by Lee et al. (2017), or the VLSI event-generator proposed by Corradi and Indiveri (2015) could be used. While these might reduce the complexity of the sensing unit and (possibly) increase the time precision, this would come at the cost of reduced flexibility.

Using this sensor, we recorded a dataset consisting of 10 repetitions of 32 stimuli (8 different angles, every 45°, and 4 distances (200, 400, 600, 800 mm). The stimuli, i.e., the surface vibrations, were induced by tapping with the index finger on a wooden surface.

### 3.2. Algorithms

In the following section we will present five different solutions to the problem of localizing the position of a vibration induced by tapping on a rigid surface. Not all approaches are entirely successful but, nonetheless, we hope to provide interesting concepts and ideas which try to emphasize how to extract task relevant information from the timing of incoming events. We selected these algorithms to represent varying levels of complexity and biological plausibility as well as because they each require different amounts of information. Specifically, in section 3.2.1 we will first demonstrate how to localize the position of the tap analytically if the geometry of sensory array and the propagation speed are known. Then in sections 3.2.2 to 3.2.6, we will present more and more biological plausible implementations which try to solve the task with less and less external information.

While these algorithms do not represent a complete list of possible solutions, we still hope to provide the reader with a thorough analysis of several approaches for computation based on the precise timing of spikes as well as outlining some of the challenges the community needs to overcome to perform such computation using event-driven SNNs. More importantly however, we hope to provide a starting point for the development of novel algorithms, as well as providing a benchmark task for further comparison and evaluation.

#### 3.2.1. Analytic Solution

**Figure 3:**
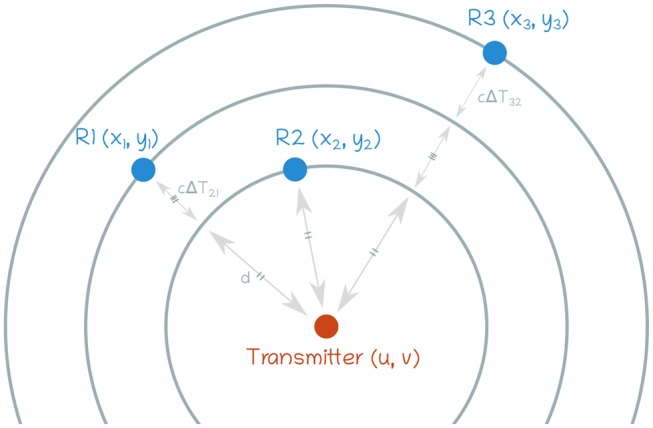
Definition of the problem. For the sake of simplicity, only 3 sensors are shown here. The transmitter can be seen as being in the center of concentric circles.

The position of the source can be estimated based on the Time Difference Of Arrival (TDOA) between each pair of sensors. The 2D problem is shown in Figure 3 and, given the sensor spatial positions *R*_*i*_(*x*_*i*_, *y*_*i*_) and the TDOA for each pair of sensors, the source localization (*u, v*) and the propagation speed *c* in the chosen medium can be retrieved as follows:

(1) $$\begin{array}{r} \underset{A}{\underset{︸}{\left[\begin{array}{cccc} x_{1}-x_{2} & y_{1}-y_{2} & -\Delta T_{12} & -\Delta T_{12}^{2}\\ x_{1}-x_{3} & y_{1}-y_{3} & -\Delta T_{13} & -\Delta T_{13}^{2}\\ \vdots & \vdots & \vdots & \vdots \\ x_{1}-x_{n} & y_{1}-y_{n} & -\Delta T_{1n} & -\Delta T_{1n}^{2} \end{array}\right]}}*\underset{X}{\underset{︸}{\left[\begin{array}{c} u\\ v\\ cd\\ c^{2} \end{array}\right]}}\\ =\underset{B}{\underset{︸}{\left[\begin{array}{c} x_{1}^{2}+y_{1}^{2}-x_{2}^{2}-y_{2}^{2}\\ x_{1}^{2}+y_{1}^{2}-x_{3}^{2}-y_{3}^{2}\\ \vdots \\ x_{1}^{2}+y_{1}^{2}-x_{n}^{2}-y_{n}^{2} \end{array}\right]}} \end{array}$$

for *N* sensors where *A* ∈ ℝ^*N*×4^, *X* ∈ ℝ^4×1^ and *B* ∈ ℝ^*N*×1^. This equation can then be solved using the pseudoinverse *A*^+^ of *A*. Because *A*^+^ has to be evaluated every time a stimulus is presented, we can exploit the fact that our matrices are well defined [rank(*A*) being equal to the number of columns of *A*] and therefore:

(2) $$\begin{array}{l} A^{+}={\left(A^{T}A\right)}^{-1}A^{T} \end{array}$$

allowing us to minimize the required computation and find an analytic solution to the problem, using bloc decomposition for the inverse (*A*^*T*^*A*)_−1_, given the fact that *A*^*T*^*A* is a squared matrix (*A*^*T*^*A* ∈ ℝ^4×4^). An alternative approach would be to iteratively estimate the pseudoinverse, following the method described by Tapson and van Schaik (2013).

#### 3.2.2. Temporal Coincidence Detection

A simple way to detect a particular position is to have a have a neuron associated with every target position, connected to each receptor with delayed synapses. In this set-up, each neuron receives one spike from every receptor and must only spike if the input came from the right place. The natural way to ensure that the receptive neuron will indeed cross the threshold is to have the spikes arrive at the same time, so that all the incoming spikes coincide and create a large increase in membrane potential.

Specifically, a decoding neuron at position *p* has *N*_*s*_ synapses, each with a corresponding delay *d*_*p*(*k*)_, and parameters τ and θ, corresponding to the decay constant and the firing threshold of the neuron. For all input spikes to arrive simultaneously, we must associate the vibration wave generate at each position *p* to a delay vector ${\text{d}}_{\text{p}}\in {\mathbb{R}}^{N_{s}}$. The sub-threshold membrane potential of the decoding neuron is then

(3) $$\begin{array}{l} v_{p}\left(t\right)=\overset{N_{s}}{\underset{k=1}{\sum }}e^{-\frac{t-t\left(k\right)-d_{p}\left(k\right)}{\tau }}\Theta \left(t-t_{k}-d_{p}\left(k\right)\right) \end{array}$$

where the exponential corresponds to the decay of the membrane potential and Θ is a step function that ensures that the input is only relevant after it arrived at the detector neuron at time *t*(*k*) + *d*_*p*_(*k*), where *t*(*k*) is the time of arrival of the ground vibration at the detector *k* and *d*_*p*_(*k*) is the delay associated with synapse *k*. The leaky integrate-and-fire neuron will spike, indicating a stimulus at position *p*, if

(4) $$\begin{array}{l} v_{\max}>\theta ,\;v_{\max}=\underset{t}{\max}v_{p}\left(t\right). \end{array}$$

Hence, we will try to maximize the value of *v*_max_. Since the exponential decay term in Equation (3) implies that each input spike is strongest at its arrival so, if we want to maximize the membrane potential, we must make sure that all those spikes arrive simultaneously, which can be achieved by setting

(5) $$\begin{array}{l} d_{p}\left(k\right)=d_{p}^{*}-t_{p}\left(k\right) \end{array}$$

where *t*_*p*_(*k*) is the *k*th entry of the vector ${\text{t}}_{p}\in {\mathbb{R}}^{N_{s}}$ which corresponds to time of arrival of the ground vibration from position *p* to each sensor *k*, and $d_{p}^{*}$ is a value that ensures that all the transmission delays are positive. Naturally, there are many possible values of $d_{p}^{*}$, but for simplicity we will set

(6) $$\begin{array}{l} d_{p}^{*}=\underset{k}{\max}\;t_{p}\left(k\right), \end{array}$$

which implies that all the spikes arrive when the last spike is detected—as it is impossible to receive all of them earlier than that.

In the ideal case—where the spikes from a given position are perfectly timed—θ = *N*_*s*_, so that, when the spikes arrive exactly at *t*_*k*_, they will all add up and the membrane potential will cross the threshold. However, if the spike times vary even slightly, the fast decay will result in a sub-threshold membrane potential and the neuron would not fire.

In the real world, the ideal case is unlikely so we must account for the possibility of jitter by associating a target detector with an area rather than a point. Assuming that τ is fixed, we must simply select the value of θ that minimizes our classification error

(7) $$\begin{array}{l} e=\text{Pr}\left[v_{\max}<\theta |p\right]+\text{Pr}\left[v_{\max}>\theta |\neg p\right], \end{array}$$

which is simply the sum of the probabilities of false negatives and false positives. The simplest way to do this is to realize that Pr[*v*_max_ < θ|*p*] increases monotonically with θ, while Pr[*v*_max_ > θ|¬*p*] decreases monotonically with θ. Hence, the computation of the optimal θ from a sample of *m* examples, where *m*_*p*_ examples were from position *p* and *m*_¬*p*_ were not, can be done through a simple algorithm:

    **for** all *m* examples **do**

        compute *v*_max_

        **if** example position = *p***then**

            add the tuple (*v*_max_, *d* = 1) to list *L*

        **else**

            add the tuple (*v*_max_, *d* = −1) to list *L*

        **end if**

    **end for**

    Sort *L* by *v*_max_, high to low.

    *e* ← *m*_*p*_

    **for** every tuple in *L*: **do**

        *e* ← *e* − *d*

        Add the tuple (*v*_max_, *e*) to list *M*

    **end for**

    select the tuple with lowest *e* in *M*

    Set θ ← *v*_max_ from the tuple with lowest error

This approach gives us a simple way of using the leaky integrate-and-fire nature of neurons to achieve the desired detection as long as we can compute the appropriate delays *a priori*.

#### 3.2.3. Complex Weights and Delays

The previous approach, while fundamentally correct, requires precise knowledge of the delays. If sensors or synapses have systematic measurement errors or there is significant jitter, it could be impossible to find delays *d*_*p*_(*k*) that would be able to fully compensate for the effects of noise. Furthermore, unreliable sensors or synapses should be given less importance than if perfect noiseless sensors or synapses. In this section we present a statistical method for computing the delays and associated weights to address this issue (State, 2019).

First we must redefine our leaky integrate and fire neuron model, described in Equation (13), to include synaptic weights:

(8) $$\begin{array}{l} v_{p}\left(t\right)=\overset{N_{s}}{\underset{k=1}{\sum }}w_{p}\left(k\right)e^{-\frac{t-t\left(k\right)-d_{p}\left(k\right)}{\tau }}\Theta \left(t-t_{k}-d_{p}\left(k\right)\right), \end{array}$$

While our new synapses now have two parameters (*w*_*p*_(*k*) and *d*_*p*_(*k*)), the logic from the previous section remains the same and our goal is to force spikes to arrive as synchronously as possible. In order to manipulate the spike times algebraically, we encode the input spike train – here consisting of a single spike per neuron – into *N*_*s*_ variables that can be studied using linear algebra. We do this by encoding spikes as phases of a complex number so each spike

(9) $$\begin{array}{l} s\left(k\right)=e^{\frac{j\pi \left(t\left(k\right)-t_{0}\right)}{T}} \end{array}$$

where *t*_0_ is the time at which the first spike of a wave arrives (so that the time of the input wave is not considered) and *j* is the imaginary unit. Encoding time in the phase of a complex number is a known trick when dealing with spikes, often used in *phasor* networks (Hirose, 1992; Reichert and Serre, 2013; Frady and Sommer, 2019). *T* is the maximum time during which we can receive spikes and is given by

(10) $$\begin{array}{l} T=\frac{2r_{\max}}{c} \end{array}$$

where *c* is the wave speed and *r*_max_ is the radius of the sensors, meaning that the numerator is two times the maximum distance between two sensors. The value of *T* ensures that the phase of *s*(*k*) is in the interval [0, π], which is necessary to avoid geometric inconsistencies (State, 2019).

Now we can use least squares regression to obtain the delays and weights associated to each synapse. Thus, for every position *p* we will have

(11) $$\begin{array}{l} \epsilon =\frac{1}{N_{e}}\overset{N_{e}}{\underset{i=1}{\sum }}||y_{i}-\overset{N_{s}}{\underset{k=1}{\sum }}{\hat{w}}_{p}\left(k\right)s_{i}\left(k\right)|{|}^{2} \end{array}$$

where *N*_*e*_ is the number of examples, indexed over *i* and *y*_*i*_ corresponds to the desired output of the perceiving neuron: one if the spikes were generated by a tap at position *p* and zero if the spikes come from a tap somewhere else.

Once we find the weight vector for position *p*, ${\hat{\text{w}}}_{p}$ = [$\hat{w}$_*p*(1)_, $\hat{w}$_*p*(2)_, …, $\hat{w}$_*p*(*n*)_], it will give us weights with complex entries. Naturally, this is not something we can put on a synapse, but rather a complex number that somehow relates *s* to its appropriate synapse. To obtain the delays and weights, we inverse the operation done in Equation (9) and obtain the delay from the phase and the weight from the absolute value,

(12) $$\begin{array}{l} {\hat{w}}_{p}\left(k\right)\to w_{p}\left(k\right)=|{\hat{w}}_{p}\left(k\right)|,\;d_{p}\left(k\right)=\frac{T}{\pi }\arg{\hat{w}}_{p}\left(k\right). \end{array}$$

It is worth noticing that the conversion from a complex weight to a weight and a delay used here ensures that all the weights are positive. This means that all synapses are excitatory, and is a natural consequence of the encoding chosen originally in Equation (9).

To understand this procedure, it is useful to look at value of ϵ. When *y*_*i*_ = 1, the sum of the input to the neuron $\overset{n}{\underset{k=1}{\sum }}\hat{w}\left(k\right)s_{i}\left(k\right)$ should be real and positive whereas, when *y*_*i*_ = 0, it should be close to zero. In the ideal case, $\hat{w}$(*k*) will have exactly the same phase as *s*_*i*_(*k*) but the opposite sign, meaning that the product $\hat{w}$(*k*)*s*_*i*_(*k*) must be real and positive and the phases somehow uniformly distributed in [0, 2π] when *y*_*i*_ = 0 so that $\overset{n}{\underset{k=1}{\sum }}\hat{w}\left(k\right)s_{i}\left(k\right)$ adds up to zero. Just as the delays were converted into phases in Equation (9), the phases must now be converted back into delays so that, when the phases of $\hat{w}$_*p*_(*k*) and *s*(*k*) cancel each other, the delay of the synapse also cancels out the delay of the spike. The weights are also easy to interpret: the more reliable the value *s*(*k*) is for a certain position *p*, the higher |$\hat{w}$_*p*_(*k*)|. This is because the least squares regression will “learn” that every time *s*(*k*) has a specific value and the product $\hat{w}$_*p*_(*k*)*s*(*k*)—which is already real and positive due to the phase cancellation—should approach *y* = 1 and hence be large.

As in the previous section, this complex conversion trick is simply a way to synchronize the arrival of spikes at the neuron encoding position and therefore we still need to compute the θ_*p*_ for every neuron, for which we can, again, use Algorithm 3.2.2. It is also worth noticing that using the complex formulation intrinsically assumes that the spikes have the shape of a cosine, as opposed to a decaying exponential (Reichert and Serre, 2013; State, 2019), meaning that it is more appropriate to use a non-instantaneous synapse such as an EPSP (Takagi, 2000) with a flat value at the maximum such that the first derivative is the same; however, this does not affect our results.

The advantage of this approach compared to similar complex formulations (Reichert and Serre, 2013; Shrestha and Orchard, 2018) lies in the use of classical linear algebra. Besides being very data efficient—as a single example would yield a solution just as well as a combination of examples—this approach easily handles cases where spikes are unreliable (State, 2019), something that is often difficult when using delays directly and it is resistant to over-fitting because the pseudoinverse guarantees that the weights will have the lowest possible modulus. However, its simplicity also make it less flexible, as it does not deal with multi-spike problems (Taherkhani et al., 2015; Shrestha and Orchard, 2018) nor does it work for SNNs with hidden units (Hirose, 1992; Frady and Sommer, 2019) as the linear algebra solution requires specific values as outputs, rather than step-by-step error feedback.

#### 3.2.4. Temporal Difference Encoders

The approaches presented in the previous sections encode target position using individual neurons to represent each point in space. While this encoding allows for precise localization, it requires a large number of neurons. In this section, we will take inspiration from the ring-like neural structures present in the sand scorpion (Stürzl et al., 2000) to develop an alternative solution based on Temporal Difference Encoder (TDE) neurons (Milde et al., 2018) which requires many fewer neurons^3^.

**Figure 4:**
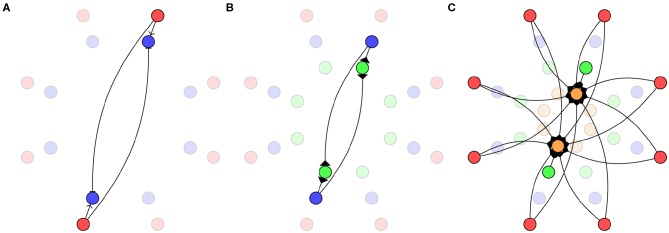
Biomimetic network architecture. **(A)** Connections from sensors (red) to TDE neurons (blue). **(B)** Connections from TDE to inverse direction neurons (green). **(C)** Connections from sensors and inverse direction neurons to direction neurons (orange).

In this model, each pair of opposite sensors is connected to an inner ring of 8 TDE neurons (Milde et al., 2018) as shown in Figure 4A. The sub-threshold behavior of the TDE neurons is modeled as a leaky integrate-and-fire neuron:

(13) $$\begin{array}{l} \tau_{m}\cdot \frac{dV\left(t\right)}{dt}=E_{L}-V\left(t\right)+R_{m}\cdot I\left(t\right) \end{array}$$

where *E*_*L*_ denotes the resting potential of the neuron, *R*_*m*_ is the membrane resistance, *I*(*t*) is the injected current at time t, and τ_*m*_ is a decay constant. These neurons are then driven by an input current *I*(*t*) such that:

(14) $$I(t)=\{\begin{array}{ll} I_{trig}\cdot f & \text{if }f>0\\ 0 & \text{otherwise} \end{array}$$

where *f* represents a dimensionless “facilitating” input and *I*_*trig*_ represents the “trigger” input current. Both *f* and *I*_*trig*_ are exponentially shaped such that:

(15) $$\begin{array}{l} \tau_{syn}\cdot \frac{df}{dt}=-f\;\tau_{syn}\cdot \frac{dI_{trig}}{dt}=-I_{trig} \end{array}$$

where τ_*syn*_ is the time constant of the synaptic dynamics. When a spike is received at a facilitating or trigger synapse, the synaptic weight (*w*_*fac*_ and *w*_*trig*_ respectively) is added to the appropriate input:

(16) $$\begin{array}{l} f\leftarrow f+w_{fac}\;I_{trig}\leftarrow I_{trig}+w_{trig} \end{array}$$

The dynamics described by Equations (14)–(16) result in an input current which is scaled non-linearly depending on the time difference between spikes arriving at the facilitating and trigger synapses. Additionally, as *I*_*trig*_ is “gated” by *f*, these synapses are also direction-selective. The time difference between the inputs received at the two opposite sensors will be largest when a stimulus is located on the line connecting them and smallest when it is on the line perpendicular to this meaning that the inner ring of TDE neurons will encode the direction of the stimuli as a vector in an over-complete 8-dimensional space.

While the directional information required could be decoded from the activity of the TDE neurons, the desired output for this system is a ring with a single active neuron identifying the direction of the stimuli. In order to achieve this, we connect the TDE neurons to a second ring of “inverse direction” neurons using the excitatory connections shown in Figure 4B. Weak connections from the TDE neuron to the adjacent inverse direction neuron and strong connections to the opposite inverse direction neuron result in this population of neurons having a minimum of activity in the direction of the stimulus.

Finally, the inverse direction neurons are connected to the innermost ring of “direction neurons” with inhibitory connections as shown in Figure 4C. These neurons are additionally provided with background excitation—direct from the sensors—tuned to produce a “1-hot” encoding of the stimuli direction. In order to maximize the accuracy of this encoding, we use 16 neurons in this ring with inhibitory weights calculated as:

(17) $$\begin{array}{l} w_{ij}=w_{peak}.max\left(0,cos\left(\theta_{i}-\phi_{j}\right)\right) \end{array}$$

where *w*_*peak*_ is the peak inhibitory weight, θ_*i*_ is the angle of the sensor adjacent to inverse direction neuron *i* and ϕ_*j*_ is the angle of the direction neuron *j*. While this approach does not currently provide an estimate of distance, if magnitude information were available, this could be provided in place of the excitatory input to the direction ring.

#### 3.2.5. Synaptic Delay Plasticity

So far we addressed how one can adjust neuronal (Θ), axonal and synaptic (*w*, τ) parameters if the geometry of the sensor array is known or, in the absence of that information, if a set of training examples is given. Given this information, we have outlined how these parameters can be optimized so that one can localize the position of a vibration source even in the presence of temporal jitter. While we used biologically motivated neuron and synapse models to perform the *computation*, the *optimization* of the parameters was done offline using conventional regression algorithms such as the least square method. Such optimization procedures require non-local information^4^ such as the neuronal firing thresholds of other neurons, the onset of the stimulus and the position of the stimulus itself. The decision on where the tap originated from is being made through adapting neuronal firing/spiking thresholds such that the designated spatio-temporal pattern is matched. In the subsequent sections we are going to address the localization task by applying three constraints on our model:

1. Only information which is local to a given pre-post synaptic neuron pair is used to update synaptic parameters.
2. No a priori knowledge of the sensory system is required.
3. The model parameters must be updated in an unsupervised manner.

Drawing inspiration from the myelin plasticity discussed in section 2.3 and from previous work on delay shifts (Hussain et al., 2012; Wang et al., 2015), in this section we will extract temporal features by modulating conduction delays.

The proposed model uses gradient descent dynamics to synchronize spikes emitted by pre-synaptic neurons, by adjusting delays on the most recently active synapses within an experimentally set temporal window. Whenever a neuron fires, mutual inhibition is used to ensure that neurons specialize to a particular temporal pattern.

The delay plasticity model works in conjunction with leaky integrate-and-fire (LIF) neurons described in Equation (13). We chose an exponential excitatory post-synaptic current (EPSC) shape such that the input current *I*(*t*) at time *t* is:

(18) $$\begin{array}{l} I\left(t\right)=I_{inj}\cdot \underset{i}{\sum }w_{i}\cdot e^{-\frac{t-s_{i}}{\tau_{syn}}}\cdot H\left(t\right) \end{array}$$

where *I*_*inj*_ is the injected current every time a neuron fires, *w*_*i*_ is the synaptic weight of synapse *i*, τ_*syn*_ is the synaptic time constant, and H(t) is the Heaviside step function.

When we study the dynamics of a single synapse *i*, we remove the discontinuities caused by the input signal by focusing on the range [*s*_*i*_, *t*] where H(t)=1, *s*_*i*_ being the time of arrival of a spike to a post-synaptic neuron. Assuming initial conditions such that *V*_*i*_(*s*_*i*_) = *E*_*L*_ as we are restricting the network to only one spike per synapse, and the membrane potential is reset between each training example through a (WTA) algorithm, the membrane equation now has a solution:

(19) $$\begin{array}{l} V_{i}\left(t\right)=E_{L}+\frac{R_{m}\cdot I_{inj}\cdot w_{i}\cdot \tau_{syn}}{\tau_{m}-\tau_{syn}}\cdot \left(e^{-\frac{t-s_{i}}{\tau_{m}}}-e^{-\frac{t-s_{i}}{\tau_{syn}}}\right) \end{array}$$

The time course of the potential follows a bi-exponential model with a finite rising time. In order to maximize the membrane potential of a post-synaptic neuron—and ultimately associate it with a particular temporal pattern—we compute the gradient of the neuron's potential $\frac{\partial V\left(t,s_{i}\right)}{\partial s_{i}}$ and modulate *d*_*i*_ until all spikes are aligned. The model assumes only one spike per synapse. The partial derivative of *V*(*t, s*_*i*_) with respect to *s*_*i*_ can then be written as:

(20) $$\begin{array}{l} \frac{\partial V\left(t,s_{i}\right)}{\partial s_{i}}=\frac{R_{m}\cdot I_{inj}\cdot w_{i}}{\tau_{m}-\tau_{syn}}\cdot \left(e^{-\frac{t-s_{i}}{\tau_{m}}}-e^{-\frac{t-s_{i}}{\tau_{syn}}}\right) \end{array}$$

The delay update rule can be represented by the following equation:

(21) $$\begin{array}{l} d_{i}^{t+1}=d_{i}^{t}+\eta \cdot \frac{\partial V\left(t,s_{i}\right)}{\partial s_{i}} \end{array}$$

where η represents the learning rate of a neuron, with η > 0. We decay the learning rate across iterations to avoid large gradient steps.

#### 3.2.6. Structural Plasticity

In this section we propose a neurally implemented, self-organizing, balanced network of excitatory and inhibitory (EI) neurons which fulfills the constraints outlined at the beginning section 3.2.5 by combining event-based STDP (Song et al., 2000) with a variant of structural plasticity (Bekkers, 2011; Knoblauch et al., 2014) and adaptive spiking thresholds (Afshar et al., 2019b). It has been demonstrated that the combination of STDP and a competitive EI network (i.e., WTA), in the context static inputs can account for disparity selectivity (Chauhan et al., 2018), in the context of non-static inputs account for the observed development of orientation selectivity (Masquelier, 2012), and even the formation of temporal memory (Kappel et al., 2014). These mechanisms become especially powerful when sensory information is encoded using relative latency, i.e., using a temporal code.

The EI network consists of N neurons of which 80 % are excitatory and 20 % are inhibitory^5^. N depends on the desired spatial resolution the network should be able to decode. Each excitatory neuron is connected via simple alpha synapse (Rall, 1967) with the following dynamics

(22) $$\begin{array}{l} I_{syn}\left(t\right)={\bar{I}}_{syn}\frac{t-t_{0}}{\tau_{syn}}\exp\left(1-\frac{t-t_{0}}{\tau_{syn}}\right) \end{array}$$

where *I*_*syn*_ is the excitatory post-synaptic current (EPSC), $\hat{I}$_*syn*_ is the peak EPSC and τ_*syn*_ is the synaptic time constant. Given a pre-synaptic spike at *t*_0_, *I*_*syn*_ is updated as follows

(23) $$\begin{array}{l} I_{syn}=I_{syn}+w, \end{array}$$

where *w* is the synaptic weight which is modified according to a STDP protocol:

(24) $$\Delta w=\{\begin{array}{l} a_{w}^{+}\cdot e^{\frac{t_{pre}-t_{post}}{\tau^{+}}},\;if\;t_{pre}\leq \;t_{post}\\ a_{w}^{-}\cdot e^{\frac{t_{pre}-t_{post}}{\tau^{-}}},\;if\;t_{pre}>\;t_{post}, \end{array}$$

where $a_{w}^{+}$ and $a_{w}^{-}$ represent the magnitude of increments and decrements to the weight and can be seen as the learning rate of the plasticity mechanism. *t*_*pre*_ and *t*_*post*_ are the times at which the pre- and post-synaptic neuron emitted a spike and τ^+/−^ defines the temporal window within which spikes result in weight changes. Such “additive” STDP updates (Song et al., 2000) often result in bimodal weight distributions with all weights ending up either at 0 or their maximum value. There are numerous approaches to addressing this problem (Goodhill and Barrow, 1994; Morrison et al., 2008), but here we implement an event-driven weight decay which is triggered whenever the post-synaptic neurons emits a spike. The weight decay depends on the synaptic weight and is calculated as follows:

(25) $$\begin{array}{l} w=w-\left(w\cdot \kappa \cdot \eta \right), \end{array}$$

where κ is the ratio between weight increment and decrement ($\kappa =\;\frac{a_{w}^{+}}{a_{w}^{-}}$, for balanced STDP protocol κ = 1) and η is the decay rate (η < <1). The objective of the STDP learning rule is to detect spatio-temporally correlated activity in the input spike trains. Here we pair STDP for synaptic weights with a STDP rule for synaptic time constants. This plasticity rule aims to find a set of synaptic time constants which, given the post-synaptic activity, increase the overlap in synaptic input currents across the 8 input channels. The time constants of channels which transmit spikes early in the sequence are increased, whereas the time constants of channels which transmit spikes late in the sequence are decreased. This plasticity rule has the consequence that a neuron spikes as early as possible to a given spatio-temporal pattern given the provided competition of the other neurons in the EI network. The synaptic time constants τ are updated as follows:

(26) $$\begin{array}{l} \Delta \tau =a_{\tau_{syn}}\cdot \frac{(\Delta t-s)\cdot e^{-\left\|\frac{\Delta t-s}{\tau^{*}-s}\right\|}}{(\tau^{*}-s)\cdot e^{-1}}, \end{array}$$

where *a*_τ_ is the learning rate of the synaptic time constant and is set such that the time scale of changes in the synaptic time constants is much slower than the time scale of weight plasticity ($a_{\tau }<<a_{w}^{+/-}$). Δ*t* is calculated based on pre- and post-synaptic spike timing (Δ*t* = *t*_*pre*_ − *t*_*post*_), τ^*^ determines the peak in change of the time constant with relative to the offset *s* (τ^*^ > *s*).

**Figure 5:**
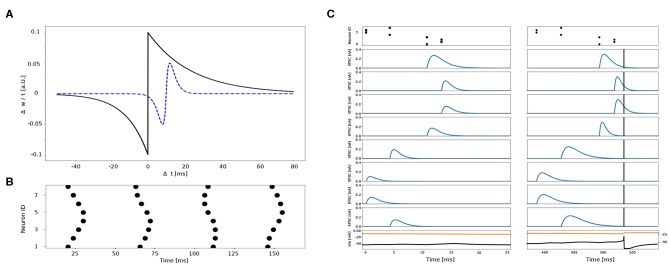
**(A)** Kernels of plasticity. Change in synaptic weights Δ*w* (black, solid) and time constants Δτ (blue, dashed) as a function of Δ*t*. **(B)** Four example spatio-temporal patterns elicited by eight different sensors. **(C)** Example synaptic traces to the same spatio-temporal pattern before (left) and after (right) training. Top panel shows the spatio-temporal pattern across eight channels. Blue traces show the excitatory post-synaptic currents (EPSCs) of 8 different synapses connected to the 8 sensors. Black vertical bar indicates the post-synaptic spike time. Black trace in the bottom panel is the post-synaptic membrane potential plotted in conjunction with its threshold (orange trace on top).

The plasticity kernel for synaptic weights and time constants are depicted in Figure 5C.

The continuous changes to synaptic weights and time constants are combined with a form of structural plasticity, operating on slower time-scale. If the average weight of all incoming synapses is below a user defined threshold, this rule deletes all (8) synapse and “re-spawns” a new set with a random weights and time constant sampled from a Gaussian distribution. This can be interpreted as modeling the retraction of a dendritic branch and its replacement by another at a different location hence its different time constant and strength (Zito et al., 1999). In this way, the synaptic weights and time constants are sampled and afterwards adjusted according to Equations (24) and (26) until each excitatory neuron within the EI network finds a unique set of *w* and τ_*syn*_. Thus, each neuron is sensitive to particular spatio-temporal pattern which, in the case of this task, represents a particular location (see Figure 5C). To prevent each neuron from learning multiple patterns we install adaptive thresholds, similar to (Afshar et al., 2019b). However, the adaption of the firing threshold depends only on the post-synaptic membrane potential, which reflects the networks activity indirectly via the recurrent connections.

## 4. Results

In the following subsections, we will present the results obtained using each of the algorithms discussed in section 3.2.

### 4.1. Analytical Solution

**Figure 6:**
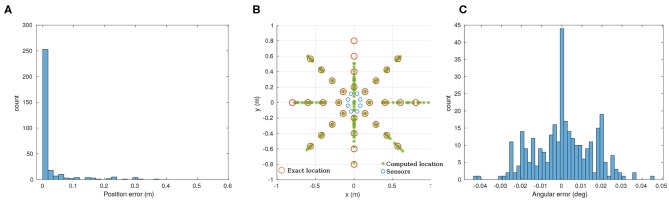
Analytic solution for the recorded data. **(A)** Position error to the ground truth. **(B)** Overview of the results for the full dataset (320 points). **(C)** Angular error. 80% of the datapoints have a position error lower than 20 mm. The angular error is lower that ±0.04 degrees. The mechanical design of our prototype presents two ill-conditioned points at 90 and −90 degrees (along a vertical line in this case).

Because the matrix *A* in Equation (1) is not constant, we need to compute its pseudo-inverse at run-time in order to use the approach presented in section 3.2.1. While it would be possible to perform this calculation on the microcontroller (either direct pseudo-inverse computation, or iterative methods), it is beyond the scope of this paper. Instead, we performed the calculation of this pseudo-inverse offline on the host computer. Due to the temporal resolution of our system, the positional error (Figure 6A) is significant (73.4% accuracy) while angles are recovered reliably in almost all trials (99.7%). Nonetheless, 80 % of the position error is less than 20 mm.

After differentiating Equation (1), it appears that our mechanical implementation has 2 ill-conditioned points, at 90 and −90 degrees, as shown in Figure 6B. As Figure 6C illustrates, the angular precision is not affected by this problem.

### 4.2. Temporal Coincidence Detection

**Figure 7:**
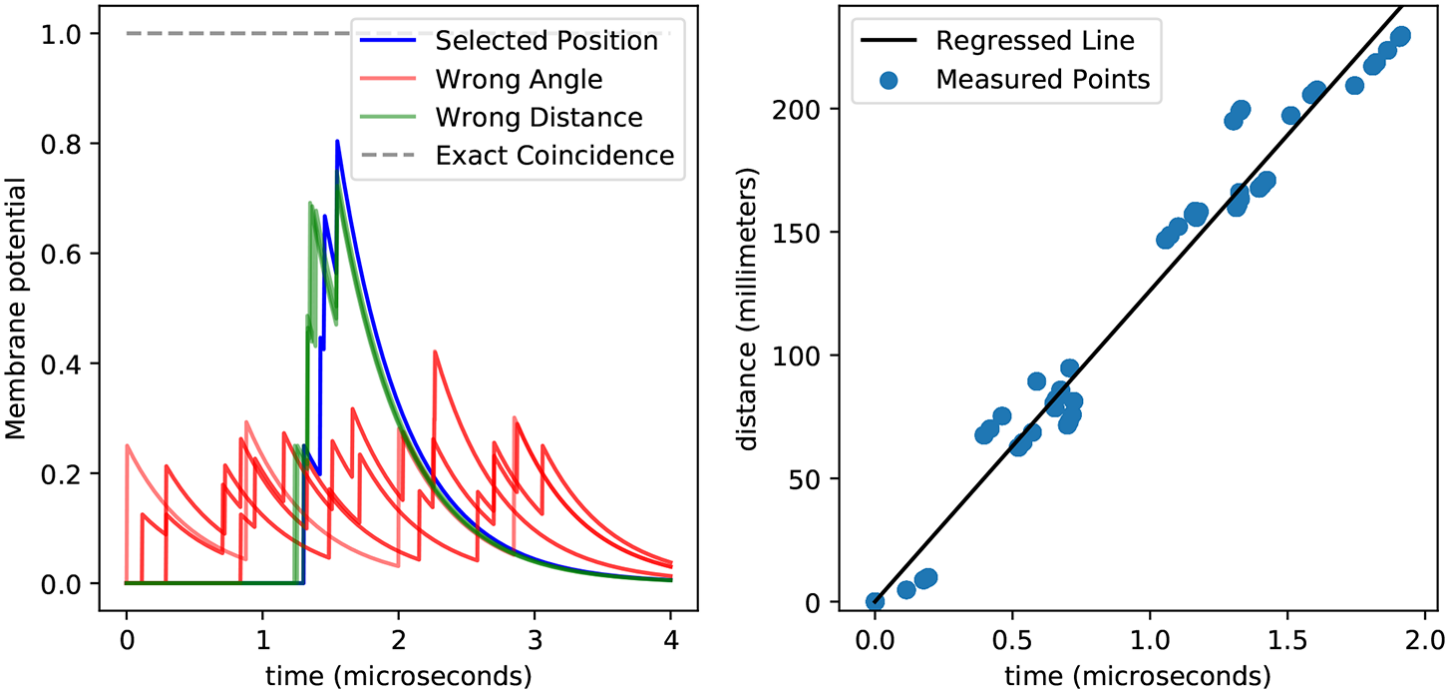
This figure shows the membrane potential for a position detector with temporal coincidence detection **(left)** and the relationship between spiking delays and distances **(right)**. The left plot shows how spikes generated from a tap at the preferred position arrive synchronously at the detector neuron and induce a high membrane potential (blue), while spikes that arrive from taps at other positions (green and red) achieve lower membrane potentials. This plot highlights the difficulty in differentiating taps coming from the same angle but different distances (green vs. blue). Also, spikes do not arrive precisely at the same time, which prevents the membrane potential from reaching the theoretical upper bound (gray). The general relationship between distance and delay is easy to see in the right plot, and can be used to obtain the speed from the slope of the regressed line (black), while the variance around that line accounts for the sub-optimal membrane potentials obtained in the left plot. It is worth noticing that here we displayed 2560 points, yet they all fall into a few dozens of clusters, meaning that the errors are systematic rather than stochastic.

To compute the delays associated with every point and sensor, we first need to calculate the propagation speed of the vibration wave on wood. The simplest way to do this is to use linear regression on the distances between points and sensors as one variable and the arrival times as the second. From our recordings we get 2.560 spike times which, as we show in Figure 7, we can match to their corresponding delay. This gives us a speed of 126 m s-1, which we can then use to compute the delays.

By using the delays calculated by dividing the distance by the propagation speed we find that we only able to recover 37.5 % of the positions successfully. Within those errors, the angles are always perfectly recovered, but the distances are not. Although the distance recovery is better than chance—which would be 25 %—the fact that the coincidence detection would mistake different distances implies that there are errors that we are not accounting for. This is not surprising given the variance around the regression line shown in Figure 7.

Interestingly, the percentage of errors remains the same when we change the number of measurement per point, hinting that the error in delays is not stochastic but rather systematic. This can be observed in Figure 7, where despite having 2,560 points we only see a few of them, meaning that the measurements are systematically biased.

### 4.3. Complex Weights and Delays

Knowing that there is a systematic but unknown bias on the sensors implies that we must resort to techniques drawn from statistics and machine learning rather than purely analytical solutions which could extract the information of the biases automatically. As expected, using the linear regression in the complex domain yields perfect recovery of the points, implying that the systematic biases in the recorded times are not necessarily an impasse.

**Figure 8:**
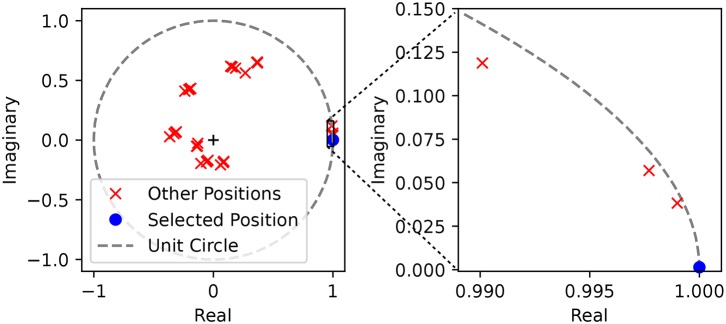
Complex representation of the spikes weighted by their corresponding complex weights. We show the position of the weighted sum of the spikes in the complex plane given by ${ŷ}_{p}=\overset{N_{s}}{\underset{k}{\sum }}{\hat{w}}_{p}\left(k\right)s\left(k\right)$ for a single position. We used the parameters of a single position detector (angle 135° and distance 400 mm), and then tested the values of ŷ_*p*_ with spike trains generated from a tap in the preferred position (blue) and in every other position (red). On the left we show the full unit circle and we observe that 32 different positions are clustered together in 8 clusters, corresponding to the 8 different angles. On the right plot we zoom around the solution and verify that the absolute value of ŷ_*p*_ for other positions within the same cluster—meaning for the same angle but different distances—is indeed lower.

This can be illustrated in Figure 8, where we see that the value obtained by the cumulative weighted representation of the spikes in the complex plane reaches the unit circle only when the right input is presented. This can be interpreted in terms of spikes by saying that for any input spike train that does not correspond to the right input the complex representation of the spikes do not have their phases aligned, and therefore they do not arrive to the perceiving neuron at the same time.

### 4.4. Temporal Difference Encoders

**Figure 9:**
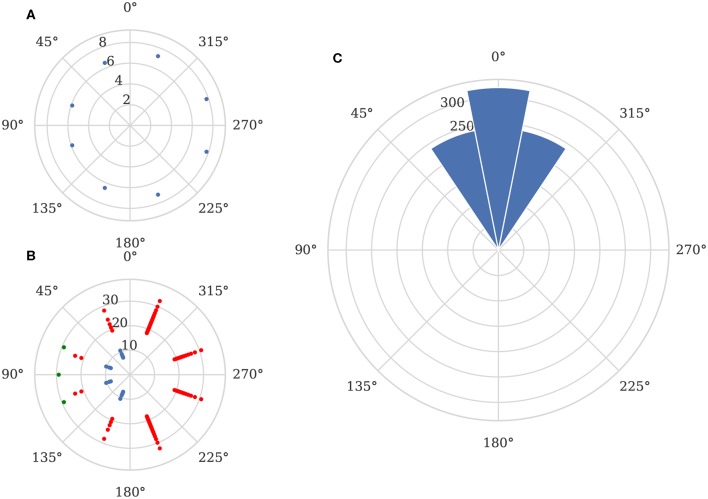
Elementary motion detector network output. **(A)** Example input spikes from sensor when stimuli is located 800 mm away from the sensor at a 90° angle. **(B)** Corresponding spikes from TDE layer (blue), inverse direction layer (red) and direction output (green). **(C)** Direction detection performance across all recorded data.

As the network described in section 3.2.4 is static, we simply presented the 320 sets of input spikes to the network as shown in Figure 9A. Because this approach is only capable of finding the *angle* to the target, we can simply treat each output spikes from a “direction” neurons (green spikes in Figure 9B) as a “vote” for the target being at the direction neuron's corresponding angle. We can then subtract the correct stimuli angle from the angle associated with each spike and plot the circular histogram shown in Figure 9C. This shows that the mean error of the classified directions is 0°, although a perfect one-hot encoding is not achieved resulting in a circular standard deviation of 17.5°.

Further investigation supports the existence of systematic biases in the sensor as, although the network is entirely symmetrical, the circular standard deviation is 0° for stimuli presented at angles of 0° and 180° whereas, for stimuli presented at all other angles, the circular standard deviation is 18.5°.

We used the GeNN library (Yavuz et al., 2016) to generate optimized CPU simulation code for this model. This simulation can be run 10 × faster than real-time on a single ARM Cortex A57 core running at 2 GHz when using a 0.1 ms simulation time step, suggesting that this approach could be used for embedded online processing of spatio-temporal patterns.

### 4.5. Synaptic Delay Plasticity

The synaptic delay plasticity network consists of eight pre-synaptic neurons, sparsely connected in a random fashion to 50 LIF neurons. Sparsity is achieved by limiting the number of connections toward a LIF neuron *N* = 4. Synaptic delays are randomly initialized according to a normal distribution with a mean of μ = 0.5 ms, and a standard deviation σ = 0.3 ms and a fixed weight equal to $\frac{w_{0}}{N}$ with *w*_0_ = 1. The resting potential is set to *E*_*L*_ = -70 mV. The LIF neuron's decay constant is set to τ_*m*_ = 2 ms, and the injected current *I*_*inj*_ is set to 180 nA to make sure that each presented spike train is capable of causing a LIF neuron to fire. The learning rate starts at η = 1 and decays by 10% after every 100 input spike trains to help the network converge toward a local minimum.

**Figure 10:**
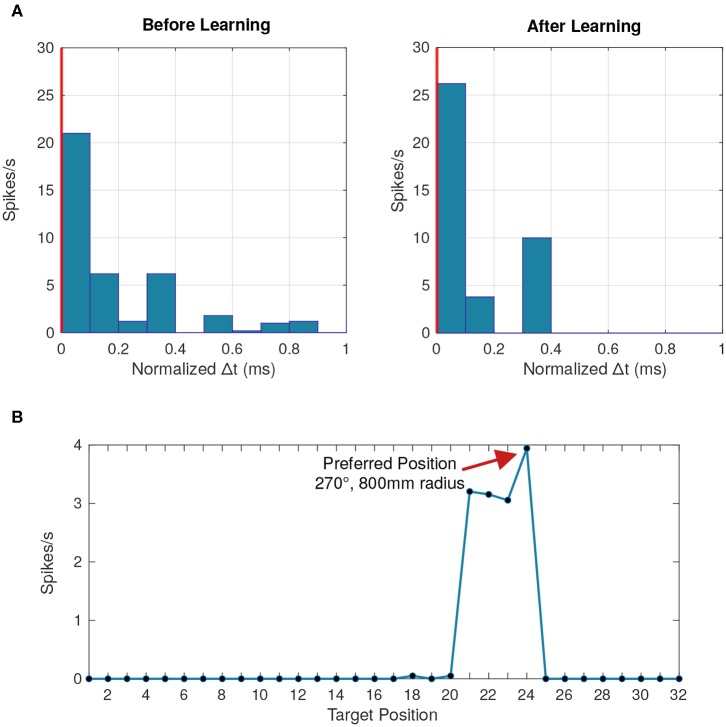
Behavior of a postsynaptic neuron with synaptic delay plasticity. **(A)** Peristimulus time histogram averaged across 50 data points at the beginning and toward the end of the simulation. The vertical red line represents the postsynaptic neuron's firing time, chosen as a reference from which the time difference is calculated. After learning, more presynaptic neurons fire with a lower time difference compared to the postsynaptic firing time, due to the synchronization of input spikes. **(B)** Tuning curve of the postsynaptic neuron averaged across all the data points where the neuron fired, linking the firing rate to each of the 32 different stimuli positions. Positions 21–24 correspond to the distances 200, 400, 600, and 800 mm, respectively, at a 270° angle.

Each post-synaptic neuron that responds starts specializing to a particular pattern by synchronizing its input spikes through a change in synaptic delays following Equation (21). The winner-take-all mechanism ensures that no other post-synaptic neurons synchronize their input spikes. With each subsequent presentation of the pattern, the time differences between input spikes gradually converge toward zero (Figure 10A).

**Figure 11:**
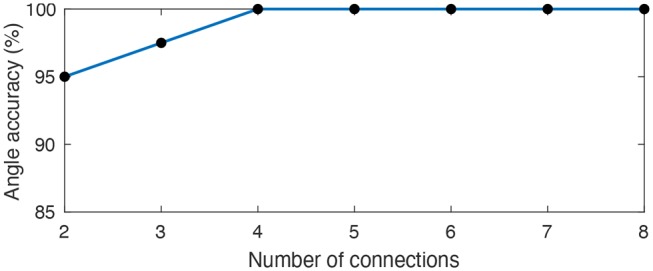
Average angle accuracy for an increasingly sparse synaptic delay network. We connected each LIF neuron to a random subset of 8 pre-synaptic neurons representing the hardware sensors. We varied the size of the subset across 35 different trials in order to assess the smallest number of connections between pre-synaptic and LIF neurons capable of preserving an accurate temporal representation of all 8 angles. A sparse synaptic delay network with only 2 connections per LIF can already represent all angles with an average accuracy of 95% and we can achieve 100% accuracy with only 4 connections.

With a temporal resolution of 0.1 ms, all eight angles were successfully represented by at least one LIF neuron (Figure 10B). Previous implementations of learned delays relied on an all-to-all connectivity scheme (Hussain et al., 2012), but we obtained similar performance with fewer connections through a randomly connected sparse network with high redundancy (Figure 11). The learned temporal patterns for a network with only two connections per LIF neuron are enough to represent all angles with an accuracy of 95% and we can achieve 100% accuracy with only four connections per LIF neuron. An all-to-all network would work just as well, but, in addition to being more efficient in terms of hardware, a randomly connected and highly redundant sparse network increases robustness against systemic noise or a faulty sensor.

We also wanted to determine whether the synaptic delay network could differentiate between stimuli at different distances. As seen in Figure 10B, an individual neuron seems to respond more frequently to a particular position. We expect the membrane potential to be maximized for a particular distance which was not the case as the membrane potential was similar across all distances.

While the delay plasticity network managed to specialize neurons to all directions, due to the slow attenuation of the waves being measured, the temporal signatures across the measured distances are not significantly different. Spike synchronization seems to have a limited impact on the membrane potential beyond a certain level of synchronization. An inhibitory plasticity rule could be explored to further specialize post-synaptic neurons to increasingly precise temporal patterns.

### 4.6. Structural Plasticity

**Figure 12:**
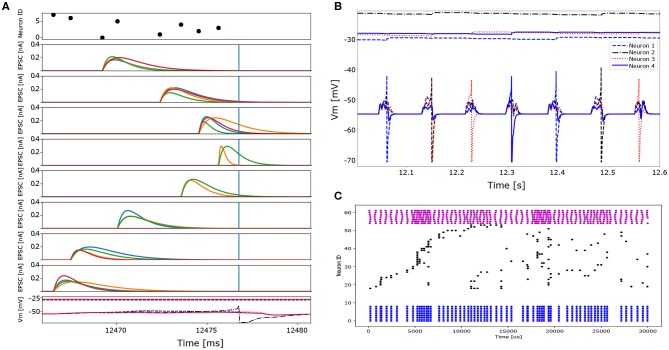
Detailed excitatory post-synaptic current traces and spiking behavior of neurons in the context of two different tasks which vary in the number of patterns presented to the network and the network size. In the first task only four different spatio-temporal patterns (four locations) are presented to a network consisting of four excitatory and 2 inhibitory neurons. In the second task 32 different spatio-temporal patterns (32 locations, i.e., the entire data set) are presented to a network of 32 excitatory and 8 inhibitory neurons. **(A)** Detailed EPSC traces of the eight input synapses to the four excitatory neurons (1 color per post-synaptic neuron) to 1 out of 4 different spatio-temporal patterns (first task). The top plot represents the input pattern, whereas the bottom panel shows the membrane potential traces of the four excitatory neurons. Note that only one neuron generates an action potential and subsequently inhibits the others via the inhibitory interneurons. **(B)** Membrane potentials and corresponding neuronal firing thresholds of 4 neurons learning 4 different spatio-temporal patterns (first task). Each neuron learns to represent a single input pattern and consequently spikes reliably to only one out of the four patterns. Two pattern repetitions are shown. After each neuron locks onto one out of the four patterns the neuronal firing threshold stabilize. **(C)** Spike raster plot of the input and the network's activity. Blue dots represent the inhibitory neuron activity (bottom), black dots indicate excitatory neuron activity (middle) and pink dots represent the different input patterns (top). The network fails to converge and represent each location using a single neuron. The reason for this might be due to the too short training time given the amount of different patterns or due to the too high similarity in the input patterns for the same angle but different distances. The sampling frequency of the ADC is too slow to provide the needed temporal precision to resolve the distance if the stimulus onset is not known.

As a first step, we trained 4 neurons to learn 4 out of the 32 different spatio-temporal patterns (see Figure 6B). The network's free parameters, i.e., synaptic weights *w*, synaptic time constants τ and firing thresholds, are randomly initialized at the beginning of the training. Each spatio-temporal pattern, corresponding to a unique location, is presented 20 times to the network. In the beginning, the neurons sparsely capture incoming spatio-temporal patterns and therefore the thresholds slowly decrease. Each neuron starts to “lock on” to one particular pattern by decreasing the synaptic time constants of late spikes in the sequence and increasing the time constants of spikes early in the sequence following Equation (26) (for visualization of the time constant change see Figure 6A, blue dashed trace). The synaptic weights start to increase more for late spikes in the sequence, than for early spikes following Equation (24) (for visualization of the weight change see Figure 6A, black solid trace). Therefore the neuron begins to respond earlier to a given spatio-temporal pattern, while the mutual inhibition introduces competition on both the spike itself and the neuronal firing thresholds. After a given neuron's threshold—and thus its other free parameters—starts to stabilize, it reliably spikes in response to a particular pattern (see Figure 12A). After 15 stimuli presentations, the thresholds start to stabilize and each neuron locks onto 1 out of 4 different patterns (Figure 12B).

In a second step, we use 32 neurons and present all 32 patterns corresponding to different locations. Each pattern is presented 100 times to the network. The neurons fail to respond reliably to the different spatio-temporal patterns which is to due to the jitter present in the data, leading to too similar spatio-temporal patterns. While neurons are capable of learning these patterns, the jitter prevents the stabilization of the firing threshold and the synaptic weights and time constants keep on changing (see Figure 12C). This case of failure might also be due to the number of presentation needed by the network to learn a unique set of parameters which scales non-linearly with the number of patterns to learn and number neurons in the network (see Afshar et al., 2014 for statistical analysis of this relation).

Structural plasticity mechanisms or variants thereof within competitive EI networks has been demonstrated before to learn spatio-temporal patterns of activity in static (Gerstner et al., 1996; George, 2018) and time-varying (Masquelier, 2012; Roy and Basu, 2016; Roy et al., 2016) conditions. Unlike Roy et al. (2016), our approach does not need a reference time, but rather relies on relative latency encoding similar to Masquelier (2012). The proposed unsupervised structural plasticity algorithm is designed to operate on time-continuous, event-based sensory data in which there exist no start- or end-point to a pattern, nor one can rely on batch-training. In contrast to the feature extraction approach proposed by Afshar et al. (2019b)—which inspired this work—neurons in our network adapt their neuronal firing threshold solely based on locally available signals. We do so by utilizing the inhibitory interneuron population which indirectly signal the presence of captured spatio-temporal pattern by other neurons by hyperpolarizing the non-spiking excitatory neurons.

Understanding the computational properties and emergent network dynamics resulting from recurrent excitation and inhibition mediated balanced activity is beyond the scope of this paper, but will be subject of future investigations. A promising next step would be to learn the temporal relations of different spatio-temporal patterns by exploiting recurrent excitatory synapses with STDP, as described in Kappel et al. (2014) and Milde (2019).

### 4.7. Comparison and Extensions

In the preceding sections of this work, we proposed several approaches for tackling the problem of spatio-temporal pattern classification, in the context of touch localization based on the precise timing of input events. Table 1 shows an overview of the presented results. It is worth noting that, as a community, we lack clear metrics for assessing the performance of spiking networks. While a simple accuracy metric can be used, it fails to consider factors such as power consumption and suitability for real-time simulation as well as not reflecting constraints present in both biological SNNs and neuromorphic hardware (Nowotny, 2014). Further effort will have to be done by the community to overcome this problem and provide datasets and metrics which *do* consider these factors.

**Table 1:** Comparison for the hereby proposed methods.

**Method**	**Angle accuracy (%)**	**Distance accuracy (%)**
Analytic solution	99.7	73.4
Temporal coincidence	100	37.5
Complex weights and delays	100	100*
Temporal difference encoders	100	N.A.
Synaptic delay plasticity	100	N.A.
Structural plasticity	100	N.A.

If all the approaches are able to distinguish between the arrival angles, the distance is still an open issue here. The analytic solution is able to (almost) correctly extract the distance, under the assumption of a known geometry. Some thoughts about this problem are detailed in the Discussion section (*). The 100% accuracy for the distance in the Complex weight and delay method is under the assumption of using an extra linear classifier to process the output data.

## 5. Discussion

In this paper we demonstrated, through a simple task, different approaches for tackling spatio-temporal pattern classification with SNNs. The problem of separating spatio-temporal patterns into prototypical features or discrete classes by learning, clustering or any other form of transformation resides at the core of both event-driven computing and event-based neuromorphic processing (Chicca et al., 2014; Indiveri and Sandamirskaya, 2019). This work is not intended to demonstrate high-precision computing, but rather to open new perspectives on learning these spatio-temporal patterns and on performing event-based tactile sensory processing. The presented algorithms were chosen to provide a qualitative overview given certain constraints of available information on how to extract task-relevant information from the timing of incoming events. Despite their different complexities, all of these approaches extract the required information solely from the precise timing of the incoming events. While a major drawback of all of these approaches is the need for temporal precision on the sensory side, our experiments expand on how information can be extracted from the timing of an incoming event in neurally-inspired processing paradigms.

As discussed in section 2.1, in sand, compressional waves attenuates rapidly with distance ($G\left(d\right)=\frac{1}{d}$) meaning that the gradient of attenuation across the scorpion's outspread legs can be used to estimate distance to the stimuli. However, on the surface used in this work, attenuation is lower. Therefore, it seems unlikely that our system would sense a difference in amplitude between the sensors. Nevertheless, *mean* amplitude across the sensors could be used to estimate distance, although it would be unable to disambiguate between a distant stimuli with a large amplitude and a nearby stimuli with a smaller amplitude.

Although it is true that the case of multiple sources is not addressed in this work, we would highlight that the precision of the sensors is of a few microseconds, meaning that the vibration should have to be generated at two sources exactly at the same time, which is unlikely. We can, however, speculate that in the case of multiple sources, the methods with excitatory synapses only—such as complex weights—should promote the activation of the neurons corresponding to the two sources, while those with lateral inhibition—such as synaptic delay plasticity—would resolve a conflict one way or the other, giving one active source at a time.

Due to the nature of the stimuli, all of the approaches presented in this paper require simulations with high temporal resolution. While small models requiring high temporal resolution—such as the TDE-based approach discussed in section 4.4—can be simulated in real-time using simple CPU-based simulations, for larger models many current approaches are not capable of providing high temporal resolution and real-time simulation speed.

The majority of digital neuromorphic systems (Furber et al., 2014; Merolla et al., 2014; Davies et al., 2018; Frenkel et al., 2018) use a time-driven approach for simulating neurons with simulation time steps of around 1 ms. While some systems can operate at a higher temporal resolution, this typically requires increasing the clock speed, leading to increased power consumption. This programmability of the SpiNNaker platform (Furber et al., 2014) means that, although this platform was *designed* to operate on a 1 ms simulation time step, it has been recently demonstrated that a 0.1 ms time step is achievable through careful programming (Knight and Furber, 2016; Rhodes et al., 2020). Furthermore, when even higher temporal resolution is required, truly event-driven models capable of learning temporal patterns with sub-millisecond precision have also been demonstrated on SpiNNaker (Lagorce et al., 2015). On the other hand, in terms of efficient processing with high temporal precision, mixed-signal analog/digital neuromorphic systems such as ROLLS (Qiao et al., 2015) or DYNAP-SE (Moradi et al., 2017) have a distinct advantage, as their neuronal dynamics arise from the physical characteristics of their analog circuits so time *represents itself* . As such, analog systems have been successfully used for a variety of complex spatio-temporal signal processing tasks, even exhibiting cognitive abilities Neftci et al. (2013), or including spike-based plasticity mechanism applied to learning auditory features from a silicon cochlea (Sheik et al., 2012), sequence learning (Kreiser et al., 2018a; Milde, 2019) and simultaneous localization and mapping using a silicon retina (Kreiser et al., 2018b).

We anticipate that this work will be extended to qualitatively and quantitatively assess solutions to the problem of spatio-temporal pattern learning, which exploit the fact that time represent itself in neural computation, and thus uses the precise timing of events to learn in a purely event-driven manner. The Neuromorphic Engineering community is facing and needs to overcome this canonical problem, to establish itself as a viable alternative to conventional clock-based sensing and processing systems.

## Data Availability Statement

The datasets generated for this study are available on request to the corresponding author.

## Author Contributions

GH and MM developed the neuromorphic tactile sensor. GH developed the analytical solution to the localization problem. PA developed the temporal coincidence detection and complex weights and delays based algorithms. JK developed the Temporal Difference Encoder based algorithm. OO developed the synaptic delay plasticity algorithm. MM developed the structural plasticity algorithm. GH, MM, PA, OO, JK, and AS wrote the paper. AS, RB, and GI contributed to the paper writing process and supervised the research.

## Conflict of Interest

The authors declare that the research was conducted in the absence of any commercial or financial relationships that could be construed as a potential conflict of interest.

## Footnotes

^1^The occurrences of examples in the data set need to be balanced.

^2^Global in this context refers to the population of feature detectors at a given level in a hierarchy, not across a hierarchy of feature detectors.

^3^The TDE was originally named the spiking Elementary Motion Detector (sEMD) as it was designed to extract relative motion cues from spatially adjacent pixels of an event-based vision sensor. However, the computation performed by these neurons is of much more general nature as it calculates the temporal correlation of two events based on the difference in timing, irrespective of the sensory modality.

^4^Local information is defined as to have direct access from a neuron perspective, e.g. post-synaptic density (estimate of synaptic weight), average firing rate (calcium concentration) or its own spiking threshold.

^5^The excitatory neuron pool has *N* × *k* incoming excitatory plastic synapses (see Equations 24 and 26), whereas the connectivity probability *E* → *I* and *I* → *E* is set to 0.7 with random and fixed synapses.
